# Two-dimensional Turbulence in Symmetric Binary-Fluid Mixtures: Coarsening Arrest by the Inverse Cascade

**DOI:** 10.1038/srep44589

**Published:** 2017-03-21

**Authors:** Prasad Perlekar, Nairita Pal, Rahul Pandit

**Affiliations:** 1 TIFR Centre for Interdisciplinary Sciences, 21 Brundavan Colony, Narsingi, Hyderabad 500075, India; 2 Centre for Condensed Matter Theory, Department of Physics, Indian Institute of Science, Bangalore 560012 India; 3 Jawaharlal Nehru Centre for Advanced Scientific Research, Jakkur, Bangalore, India

## Abstract

We study two-dimensional (2D) binary-fluid turbulence by carrying out an extensive direct numerical simulation (DNS) of the forced, statistically steady turbulence in the coupled Cahn-Hilliard and Navier-Stokes equations. In the absence of any coupling, we choose parameters that lead (a) to spinodal decomposition and domain growth, which is characterized by the spatiotemporal evolution of the Cahn-Hilliard order parameter *ϕ*, and (b) the formation of an inverse-energy-cascade regime in the energy spectrum *E(k*), in which energy cascades towards wave numbers *k* that are smaller than the energy-injection scale *kin j* in the turbulent fluid. We show that the Cahn-Hilliard-Navier-Stokes coupling leads to an arrest of phase separation at a length scale *Lc*, which we evaluate from *S(k*), the spectrum of the fluctuations of *ϕ*. We demonstrate that (a) *Lc* *~* *LH*, the Hinze scale that follows from balancing inertial and interfacial-tension forces, and (b) *Lc* is independent, within error bars, of the diffusivity *D*. We elucidate how this coupling modifies *E(k*) by blocking the inverse energy cascade at a wavenumber *kc*, which we show is ≃2*π*/*Lc*. We compare our work with earlier studies of this problem.

Binary-fluid mixtures (such as oil and water) have played a pivotal role in the development of the understanding of (a) equilibrium critical phenomena at the consolute point, above which the two fluids mix^1^,^2^,^3^, (b) nucleation^4^, and (c) spinodal decomposition, the process by which a binary-fluid mixture, below the consolute point and below the spinodal curve, separates into the two, constituent liquid phases until, in equilibrium, a single interface separates the two coexisting phases (this phase separation is also known as coarsening)^5^,^6^. In the presence of flows, the demixing because of spinodal decomposition gets arrested and an emulsion is formed. This process, also known as coarsening arrest, is important in several three-dimensional (3D) and two-dimensional (2D) turbulent flows. The former have been studied recently^7^,^8^,^9^. Coarsening arrest in a 2D, turbulent, binary-fluid mixture is also of relevance to problems such as the dynamics of oil slicks on the surface of the ocean, whose understanding is of clear socio-economic and scientific relevance^10^,^11^,^12^,^13^. Oceanic flows have been modelled successfully as 2D, turbulent fluids. Such 2D turbulence is fundamentally different from three-dimensional (3D) fluid turbulence as noted in the pioneering studies of Fjørtoft, Kraichnan, Leith, and Batchelor^14^,^15^,^16^,^17^,^18^. In particular, the fluid-energy spectrum in 2D turbulence shows (a) a *forward cascade* of enstrophy (or the mean-square vorticity), from the energy-injection wave number *k*_*inj*_ to larger wave numbers, *and* (b) an *inverse cascade* of energy to wave numbers smaller than *k*_*inj*_. We elucidate the turbulence-induced arrest of phase separation in a 2D, symmetric, binary-fluid mixture.

Coarsening arrest by 2D turbulence has been studied in ref. 19, where it has been shown that, for length scales smaller than the energy-injection scale 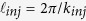, the typical linear size of domains is controlled by the average shear across the domain. However, the nature of coarsening arrest, for scales larger than 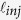, i.e., in the inverse-cascade regime, which is relevant for large-scale oceanic flows, still remains elusive. In particular, it is not clear what happens to the inverse energy transfer, in a 2D binary-fluid, turbulent mixture, in which the mean size of domains provides an additional, important length scale. We resolve these two issues in our study. By combining theoretical arguments with extensive direct numerical simulations (DNSs) we show that the Hinze length scale *L*_*H*_ (see refs 8,9) provides a natural estimate for the arrest scale; and the inverse flux of energy also stops at a wave-number scale 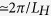. Coarsening arrest has also been studied in simple shear flows (refs 20, 21, 22, 23, 24, 25), which yield coarsening arrest with domains elongated in the direction of shear.

Forced, 2D, statistically steady, Navier-Stokes-fluid turbulence displays a forward cascade of enstrophy, from 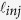 to smaller length scales, and an inverse cascade of energy to length scales smaller than 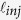. In the inverse-cascade regime, on which we concentrate here, *E(k*) ~ *k*^−5/3^ (see, e.g., refs 15,18) and the energy flux Π(*k*) ~ *ε* ≡ 〈*ε(t*)〉_*t*_ assumes a constant value. For the Cahn-Hilliard model, if it is *not* coupled to the Naiver-Stokes equation, 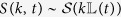, for large times, where the time-dependent length scale 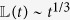, in the early Lifshitz-Slyozov^26^,^27^,^28^,^29^ regime; if the Cahn-Hilliard model is coupled to the Navier-Stokes equation, then, in the absence of forcing, 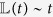, in the viscous-hydrodynamic regime, first discussed by Siggia^27^,^28^,^29^,^30^, and 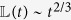, in the very-late-stages in the Furukawa^31^ and Kendon^32^ regimes. For a discussion of these regimes and a detailed exploration of a universal scaling form for 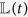 in 3D we refer the reader to ref. 33. We now elucidate how these scaling forms for *E(k*) and *S(k, t*) are modified when we study forced 2D turbulence, in the inverse-cascade regime in the coupled Cahn-Hilliard-Navier-Stokes equations.

## Results

### Cahn-Hilliard-Navier-Stokes equations

We model a symmetric binary-fluid mixture by using the incompressible Navier-Stokes equations coupled to the Cahn-Hilliard or Model-H equations^34^,^35^. We are interested in 2D incompressible fluids, so we use the following stream-function-vorticity formulation^36^,^37^,^38^ for the momentum equation:


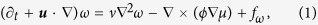



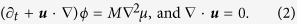


Here ***u**(**x**, t*) ≡ (*u*_*x*_, *u*_*y*_) is the fluid velocity at the point ***x*** and time *t*, 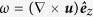, *ϕ(**x**, t*) is the Cahn-Hilliard order parameter that is positive in one phase and negative in the other, *p(**x**, t*) is the pressure, 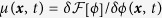 is the chemical potential, 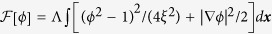 is the free energy, Λ is the mixing energy density, *ξ* controls the width of the interface between the two phases of the binary-fluid mixture, *ν* is the kinematic viscosity, the surface tension 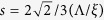, the mobility of the binary-fluid mixture is *M*, and *f*_*ω*_ is the external driving force. For simplicity, we study mixtures in which *M* is independent of *ϕ* and both components have the same density and viscosity^33^. We use periodic boundary conditions in our square simulation domain, with each side of length *L* = 2*π*. To obtain a substantial inverse-cascade regime, we stir the fluid at an intermediate length scale by forcing in Fourier space in a spherical shell with wave-number 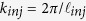. Our choice of forcing 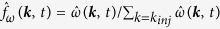, where the caret indicates a spatial Fourier transform, ensures that there is a constant enstrophy-injection rate. The higher the Reynolds number *Re* ∝ 1/*ν*, the more turbulent is the flow; and the higher the Weber number *We* ∝ 1/*σ*, the more the fluctuations in the domains (see Table 1 for definitions of *Re, We*, and other parameters in our study). To elucidate the physics of coarsening arrest, we conduct direct numerical simulations (DNSs) of Eqs (1) and (2) (see Methods Section for details).

### Coarsening Arrest

**Figure 1:**
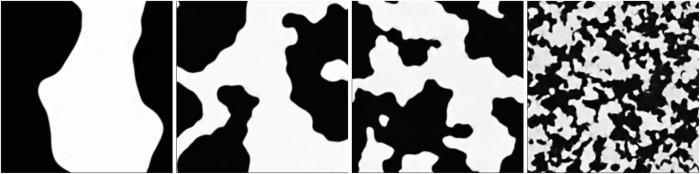
Pseudo-gray-scale plots of the order parameter field *ϕ*, at late times when coarsening arrest has occured, in 2D symmetric-binary-fluid turbulence with *Re* = 124. Note that the domain size decreases as we increase the Weber number *We* from the leftmost to the rightmost panel: *We* = 1.2 · 10^−2^ (R3); *We* = 5.9 · 10^−2^ (R4); *We* = 1.2 · 10^−1^ (R5); and *We* = 5.9 · 10^−1^ (R8).

In Fig. 1 we show pseudo-gray-scale plots of *ϕ*, at late times when coarsening arrest has occurred, for four different values of *We* at *Re* = 124; we find that the larger the value of *We* the smaller is the linear size that can be associated with domains; this size is determined by the competition between turbulence-shear and interfacial-tension forces. This qualitative effect has also been observed in earlier studies of 2D and 3D turbulence of symmetric binary-fluid mixtures^19^,^20^,^21^,^39^,^40^,^41^,^42^,^43^,^44^.

We calculate the coarsening-arrest length scale


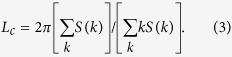


we now show that *L*_*c*_ is determined by the Hinze scale *L*_*H*_, which we obtain, as in Hinze’s pioneering study of droplet break-up^9^, by balancing the surface tension with the inertia as follows:


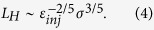


**Figure 2:**
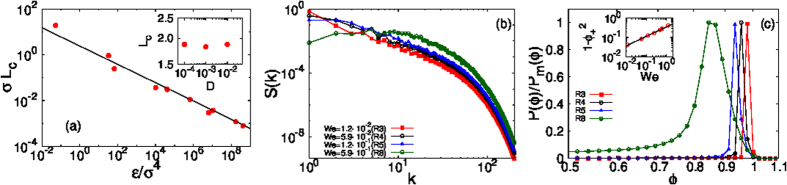
(**a**) Log-log (base 10) plot of *σL*_*c*_ versus *ε*/*σ*^4^ showing data points (*L*_*c*_ from Equation (3), with *S(k*) from our DNS) in red. The black line is the Hinze result (4) for *L*_*H*_; a fit to our data yields a constant of proportionality 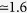 and an excellent approximation to the arrest scale *L*_*c*_ over several orders of magnitude on both vertical and horizontal axes; the plot of *L*_*c*_ versus *D*, in the inset, shows that, for fixed values of *ε*_*ν*_ and *σ* (runs R1, R2 and R4), *L*_*c*_ is independent of *D* (within error bars), as is implied by the Hinze condition (see text). (**b**) Log-log (base 10) plots of the spectrum *S(k*), of the phase-field *ϕ*, versus *k*; as *We* increases (i.e., *σ* decreases) the low-*k* part of *S(k*) decreases and *S(k*) develops a broad and gentle maximum whose peak moves out to large values of *k*. (**c**) Plots versus *ϕ*, in the vicinity of the maximum at *ϕ*_+_, of the normalized PDFs *P(ϕ*)/*P*_*m*_(*ϕ*), where *P*_*m*_(*ϕ*) is the maximum of *P(ϕ*); the peak position *ϕ*_+_ → 1 as *We* increases (see the inset which suggests that 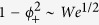 (black line)).

We obtain for 2D, binary-fluid turbulence the intuitively appealing result *L*_*c*_ ~ *L*_*H*_ (for a similar, recent Lattice-Boltzmann study in 3D see ref. 8). In particular, if we determine *L*_*c*_ from Eq. (3), with *S(k*) from our DNS, we obtain the red points in Fig. 2, which is a log-log plot of *σL*_*c*_ versus *ε*_*inj*_/*σ*^4^; the black line is the Hinze result (4) for *L*_*H*_, with a constant of proportionality that we find is 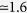 from a fit to our data. We see from Fig. 2 that the Hinze length scale *L*_*H*_ gives an excellent approximation to the arrest scale *L*_*c*_ *over several orders of magnitude on both vertical and horizontal axes*. Note that the Hinze estimate also predicts that, for fixed values of *ε*_*inj*_ and *σ*, the coarsening-arrest scale is independent of *D*; the plot of *L*_*c*_ versus *D*, in the inset of Fig. 2, shows that our data for *L*_*c*_ are consistent (within error bars) with this prediction.

In Fig. 2(b) we show clearly how the arrest of coarsening manifests itself as a suppression of *S(k*), at small *k* (large length scales). This suppression increases as *We* increases (i.e., *σ* decreases); and *S(k*) develops a broad and gentle maximum whose peak moves out to large values of *k* as *We* grows. These changes in *S(k*) are associated with *We*-dependent modifications in the probability distribution function (PDF) *P(ϕ*) of the order parameter *ϕ*, which is symmetrical about *ϕ* = 0 and has two peaks at *ϕ* = *ϕ*_±_, where *ϕ*_+_ = −*ϕ*_−_ > 0; we display *P(ϕ*)/*P*_*m*_(*ϕ*) in Fig. 2(c) in the vicinity of the peak at *ϕ*_+_; as *We* increases, *ϕ*_+_ decreases; here *P*_*m*_(*ϕ*) is the maximum value of *P(ϕ*). In particular, our DNS suggests that 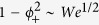, for small *We*.

The modification in *P(ϕ*) can be understood qualitatively by making the approximation that the effect of the fluid on the equation for *ϕ* can be encapsulated into an eddy diffusivity *D*_*e*_^42^,^45^,^46^. The eddy-diffusivity-modified Cahn-Hilliard equation is ∂_*t*_*ϕ* = (*D*_*e*_ − *D*)∇^2^*ϕ* + *D*∇^2^*ϕ*^3^ + *M*Λ∇^4^*ϕ*, which gives the maximum and minimum values of *ϕ* as 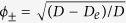. Furthermore, if we neglect the nonlinear term^27^,^29^, we find easily that the modified growth rate is *Dk*^2^[(1 − *D*_*e*_/*D*) − *M*Λ*k*^2^]; i.e., all wave numbers larger than 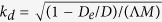 are stable to perturbations. In particular, droplets with linear size <(2*π*/*k*_*d*_) decay in the presence of coupling with the velocity field; we expect, therefore, that, in the presence of fluid turbulence, the peak of *P(ϕ*) broadens and shifts as it does in our DNS. For a quantitative description of this broadening and the shift of the peak, we must, of course, carry out a full DNS of the Cahn-Hilliard-Navier-Stokes equation as we have done here.

### Energy spectrum

We have investigated, so far, the effect of fluid turbulence on the phase-field *ϕ* and its statistical properties such as those embodied in *S(k*) and *P(ϕ*). We show next how the turbulence of the fluid is modified by *ϕ*, which is an *active* scalar insofar as it affects the velocity field. In the statistically steady state of our driven, dissipative system, the energy injection must be balanced by both viscous dissipation and dissipation that arises because of the interface, i.e., we must have *ε*_*inj*_ = *ε*_*ν*_ + *ε*_*μ*_.

**Figure 3:**
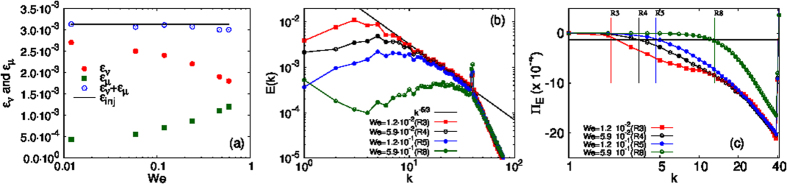
(**a**) Plots of the statistically-steady-state values of *ε*_*ν*_, *ε*_*μ*_, and their sum 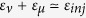 versus *We*. (**b**) Log-log (base 10) plots of the energy spectrum *E(k*) versus *k*, for different values of *We*, illustrating the truncation of the inverse energy cascade as *We* increases. The black line indicates the *k*^−5/3^ result for the inverse-cascade regime in 2D fluid turbulence. (**c**) Log-log (base 10) plots of the energy flux Π_*E*_(*k*) versus *k* for different values of *We*. The intersection of the line 0.06*ε*_*inj*_ (black line) with Π_*E*_(*k*) gives *k*_*c*_, the wave-number at which the inverse energy cascade gets truncated; our estimate of the arrest scale 2*π*/*L*_*c*_ (vertical lines) is comparable to *k*_*c*_.

In Fig. 3(a), we show that *ε*_*ν*_ decreases and *ε*_*μ*_ increases as we increase *We*, while keeping *ε*_*inj*_ constant, because *L*_*c*_ diminishes (Fig. 1) and, therefore, the interfacial length and *ε*_*μ*_ increase. This decrease of *L*_*c*_ is mirrored strikingly in plots of the fluid-kinetic-energy spectrum *E(k*) (Fig. 3(b)), which demonstrate that the inverse cascade of energy is effectively blocked at a wavenumber *k*_*c*_, which we determine below, from the energy flux, and which we find is 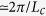, where *L*_*c*_ follows from *S(k*) (see Fig. 2). The value of *k*_*c*_ increases with *We*; and the inverse cascade is completely blocked for the largest *We* we use, for which 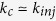, the forcing scale.

To provide clear evidence that the blocking of the energy flux is closely related to the arrest scale, we show in Fig. 3(c) plots of the energy flux 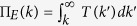 for different values of *We*. Here 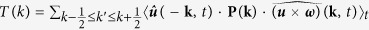 is the energy transfer and **P**(**k**) is the transverse projector with components *P*_*ij*_(*k*) ≡ *δ*_*ij*_ − *k*_*i*_*k*_*j*_/*k*^2^. We define *k*_*c*_ as the wave-number at which Π_*E*_(*k*) comes within 4% of *ε*_*inj*_. We find that the wave-numer corresponding to the arrest scale 2*π*/*L*_*c*_ (marked by vertical lines for each run) is comparable to *k*_*c*_.

**Figure 4:**
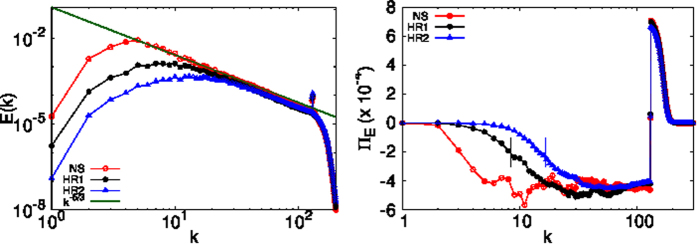
Log-log (base 10) plots of the kinetic energy spectrum *E(k*) (left) and the corresponding energy flux Π_*E*_(*k*) for our runs *HR*1:*We* = 1.7 · 10^−2^ and *HR*2:*We* = 4.3 · 10^−2^. We also plot the single-phase Navier-Stokes energy spectrum and the energy flux for reference. On increasing *We*, small domains are formed and these lead to a truncation of energy flux at a wave-number around 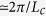 (marked by vertical lines).

In the presence of the standard viscous term *ν*∇^2^***u*** and the Ekman drag *α**u***, it is not possible to see a large range of constant energy flux^47^,^48^. However, it is possible to attain a large constant energy flux range by carrying out DNSs using hyperviscosity and hypoviscosity^47^ (see the Methods Section for details). The plot in Fig. 4(left) shows the energy spectrum and the corresponding energy flux obtained [Fig. 4(right)] from our runs *HR*1 and *HR*2. Consistent with the earlier discussion, we find that the coarsening length *L*_*c*_ decreases on increasing *We*. Furthermore, the formation of arrest-scale domains leads to a blockage of the energy cascade; because we use hypoviscosity, we now see clear evidence of a constant energy flux over a decade for the single-phase Navier-Stokes run. For the binary-fluid case, the energy flux remains constant for a shorter range and then decreases to zero around a wave-number 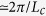.

### Passive advection

**Figure 5:**
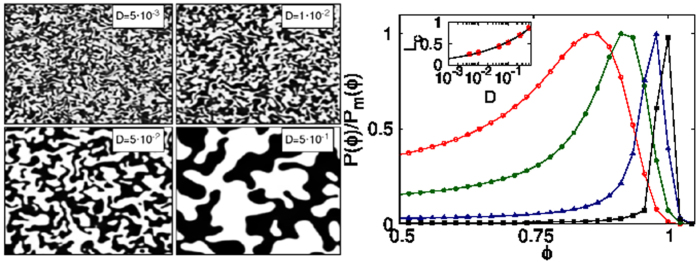
Passive-advection model: (Left panel) Pseudo-gray-scale plots of the order parameter *ϕ* for different values of the diffusivity *D* (cf. Fig. 1). (Right panel) Plots of *P(ϕ*)/*P*_*m*_(*ϕ*), in the vicinity of the maximum at *ϕ*_+_ [cf. Fig. 2(c)];the inset shows that *L*_*c*_ ~ *D*^0.27^ (black line), which is in stark contrast to the Cahn-Hilliard-Navier-Stokes result in the inset of Fig. 2(a).

It has been suggested^22^,^45^,^46^ that coarsening arrest can be studied by using a model in which the field *ϕ* is advected passively by the fluid velocity. Such a passive-advection model is clearly inadequate because it cannot lead to the phase-field-induced modifications in the statistical properties of the turbulent fluid (see Fig. 3). The passive-advection case is easily studied by turning off the coupling term *ϕ*∇*μ* in Eq. (2). We then contrast the results for this case with the ones we have presented above. The parameters we use for the passive-advection DNS are *N* = 1024, Λ = *ξ*^2^,*ξ* = 0.0176; and we carry out runs for *D* = 5 · 10^−3^, 1 · 10^−2^, 5 · 10^−2^ and 5 · 10^−1^. The evolution of the pseudo-grayscale plots of *ϕ* with *D*, in the left panel of Fig. 5, is qualitatively similar to the evolution shown in Fig. 1. There is also a qualitative similarity in the dependence on *D* of the scaled PDFs *P(ϕ*)/*P*_*m*_(*ϕ*); we can see this by comparing the passive-advection result, shown in the middle panel of Fig. 5 for positive values of *ϕ* in the vicinity of the peak, with its counterpart in Fig. 2(c). However, there is a qualitative difference in the dependence of *L*_*c*_ on *D*: in the passive-advection case we find *L*_*c*_ ~ *D*^0.27^ [Fig. 5 (inset)], which is in stark contrast to the essentially *D*-independent behavior of *L*_*c*_ shown in the inset of Fig. 2(c).

## Discussion

In conclusion, our extensive study of two-dimensional (2D) binary-fluid turbulence shows how the Cahn-Hilliard-Navier-Stokes coupling leads to an arrest of phase separation at a length scale *L*_*c*_, which follows from *S(k*). We demonstrate that *L*_*c*_ ~ *L*_*H*_, the Hinze scale that we find by balancing inertial and interfacial-tension forces, and that *L*_*c*_ is independent, within error bars, of the diffusivity *D*. We also elucidate how the coupling between the Cahn-Hilliard and Navier-Stokes equations modifies the properties of fluid turbulence in 2D. In particular, we show that there is a blocking of the inverse energy cascade at a wavenumber *k*_*c*_, which we show is 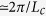.

Earlier DNSs of turbulence-induced coarsening arrest in binary-fluid phase separation have concentrated on regimes in which there is a forward cascade of energy in 3D (see ref. 8) and a forward cascade of enstrophy in 2D (see ref. 19). Although studies that use a passive-advection model for *ϕ* obtain results that are qualitatively similar to those we obtain for *S(k*) and the spatiotemporal evolution of *ϕ*, they cannot capture the phase-field-induced modification of the statistical properties of fluid turbulence and the correct dependence of *L*_*c*_ on *D*. We find our results to be in qualitative agreement with the earlier studies on the advection of binary-fluid mixtures with synthetic chaotic flows^45^,^46^; of course, such studies cannot address the effect of the phase field on the turbulence in the binary fluid.

Some groups have also studied the statistical properties of turbulent, symmetric, binary-fluid mixtures above the consolute point, where the two fluids mix even in the absence of turbulence^40^,^49^,^50^. In these studies, there is, of course, neither coarsening nor coarsening arrest.

We hope our study will lead to new experimental studies of turbulence in binary-fluid mixtures, especially in 2D^51^,^52^,^53^,^54^, to test the specific predictions we make for *L*_*c*_ and the blocking of the inverse cascade of energy.

## Methods

### Cahn-Hilliard-Navier-Stokes equations: Direct Numerical Simulations

We conduct direct numerical simulations (DNSs) of Eqs (1) and (2) by using a Fourier pseudospectral method^55^; because of the cubic nonlinearity in the chemical potential *μ*, we use *N*/2-dealiasing. For time integration we use the exponential Adams-Bashforth method ETD2^56^. To obtain a substantial inverse-cascade regime, we stir the fluid at an intermediate length scale by forcing in Fourier space in a spherical shell with wave-number 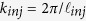. Our choice of forcing 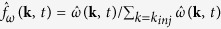, where the caret indicates a spatial Fourier transform, ensures that there is a constant enstrophy-injection rate. The parameters for our DNSs are given in Table 1.

Given ***u**(**x**, t*) and *ϕ(**x**, t*) from our DNS, we calculate the energy and order-parameter (or phase-field) spectra, which are, respectively, 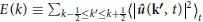 and 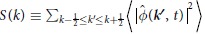, where 〈〉_*t*_ denotes the average over time in the statistically steady state of our system. The total kinetic energy is 
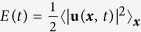
 and the total enstrophy 
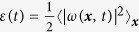
, where 〈〉_***x***_ denotes the average over space, 〈*f*_*ω*_*ω*〉 is the enstrophy-injection rate, which is related to the energy-injection rate via 
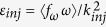
, *E* = 0.5∑_*k*_*E(k*) is the fluid kinetic energy, *ε*_*ν*_ = *ν*∑_*k*_*k*^2^*E(k*) is the fluid-energy dissipation rate, and 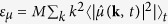 is the energy-dissipation rate because of the phase field *ϕ*.

### Hyperviscous Cahn-Hilliard-Navier-Stokes equations

The Cahn-Hilliard-Navier-Stokes equations with modified viscosity terms are^47^:


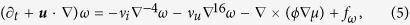



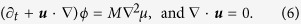


Here we use a hypo-viscosity term −*ν*_*i*_∇^−4^*ω* to dissipate energy at large scales and a hyperviscosity term −*ν*_*u*_∇^16^*ω* to dissipate enstrophy at small scales. As discussed in the main text, we use a constant-energy-injection forcing with *k*_*inj*_ = 130. The other parameters for our simulations are given in Table 2.

## Additional Information

**How to cite this article:** Perlekar, P. *et al*. Two-dimensional Turbulence in Symmetric Binary-Fluid Mixtures: Coarsening Arrest by the Inverse Cascade. *Sci. Rep.* **7**, 44589; doi: 10.1038/srep44589 (2017).

**Publisher's note:** Springer Nature remains neutral with regard to jurisdictional claims in published maps and institutional affiliations.

## Figures and Tables

**Table 1:** Parameters *N, ν, M,ξ*, Λ, *D*, 〈*f*_*ω*_*ω*〉 for our DNS runs R1-R12.

	*N*	*ν*	*M*	*ξ*(×10^−2^)	Λ(×*ξ*^2^)	*D*	〈*f*_*ω*_*ω*〉	*E*	*ε*_*ν*_	*ε*_*μ*_	*We*	*Lc*
R1	1024	10^−4^	10^−2^	1.76	1.0	10^−2^	5.0	3.3 · 10^−2^	2.5 · 10^−3^	5.7 · 10^−4^	5.9 · 10^−2^	1.87
R2	1024	10^−4^	10^−4^	1.76	1.0	10^−4^	5.0	3.1 · 10^−2^	2.6 · 10^−3^	8.1 · 10^−4^	5.9 · 10^−2^	1.87
R3	2048	10^−4^	2 · 10^−4^	1.76	5.0	10^−3^	5.0	4.8 · 10^−2^	2.7 · 10^−3^	4.3 · 10^−4^	1.2 · 10^−2^	2.97
R4	2048	10^−4^	1 · 10^−3^	1.76	1.0	10^−3^	5.0	3.0 · 10^−2^	2.5 · 10^−3^	5.6 · 10^−4^	5.9 · 10^−2^	1.82
R5	2048	10^−4^	2 · 10^−3^	1.76	5.0 · 10^−1^	10^−3^	5.0	2.3 · 10^−2^	2.4 · 10^−3^	7.1 · 10^−4^	1.2 · 10^−1^	1.35
R6	1024	10^−4^	4 · 10^−3^	1.76	2.5 · 10^−1^	10^−3^	5.0	1.5 · 10^−2^	2.2 · 10^−3^	8.7 · 10^−4^	2.4 · 10^−1^	0.9
R7	1024	10^−4^	8 · 10^−3^	1.76	1.25 · 10^−1^	10^−3^	5.0	9.5 · 10^−3^	1.9 · 10^−3^	1.1 · 10^−3^	4.7 · 10^−1^	0.57
R8	2048	10^−4^	10^−2^	1.76	1.0 · 10^−1^	10^−3^	5.0	8.1 · 10^−3^	1.8 · 10^−3^	1.2 · 10^−3^	5.9 · 10^−1^	0.48
R9	2048	10^−4^	2 · 10^−4^	0.50	5.0	10^−3^	5.0	3.7 · 10^−2^	2.8 · 10^−3^	3.4 · 10^−4^	4.1 · 10^−2^	1.55
R10	2048	10^−4^	10^−3^	0.50	1.0	10^−3^	5.0	1.7 · 10^−2^	2.6 · 10^−3^	5.3 · 10^−4^	2.1 · 10^−1^	0.63
R11	1024	10^−2^	2 · 10^−4^	1.76	5 · 10^2^	10^−1^	4 · 10^5^	2.0 · 10^1^	2.4 · 10^2^	1.2 · 10^1^	0.22	2.3
R12	1024	10^−2^	10^−3^	1.76	10^2^	10^−1^	4 · 10^5^	8.8	2.0 · 10^2^	4.7 · 10^1^	1.1	0.55

The forcing wave number is fixed at 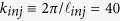, *N*^2^ is the number of collocation points, *ν* is the kinematic viscosity, *M* is the mobility parameter, *ξ* sets the scale of the interface width, the surface tension 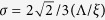, 〈*f*_*ω*_*ω*〉 is the enstrophy-injection rate, which is related to the energy-injection rate [
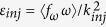
], *D* ≡ *M*Λ/*ξ*^2^ is the diffusivity of our binary-fluid mixture, *E* = 0.5∑_*k*_|*u*_*k*_|^2^ is the fluid kinetic energy, *ε*_*ν*_ = *ν*∑_*k*_*k*^2^|*u*_*k*_|^2^ is the fluid energy dissipation rate, 
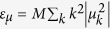
 is the energy dissipation rate because of the phase-field *ϕ*, 
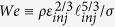
, 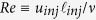 is the Reynolds number, *L*_*c*_ is the coarsening-arrest length scale [Eq. (3)]. *R*1 − *R*2: *Re* = 124 and *Sc* = 1; *R*3 − *R*10: *Re* = 124 and *Sc* = 0.1; *R*11 − *R*12: *Re* = 53 and *Sc* = 0.1. In some of our runs we also include a friction term −*αω* on the right-hand-side of Eq. (1); *α* = 0.001 for runs *R*4 − *R*8 and zero otherwise. In all our studies we use *k*_*inj*_ = 40 so that there is a clear separation between 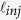 and *ξ*.

**Table 2:** Parameters *N, ν*_*u*_, *ν*_*i*_, *M,ξ*, Λ, 〈*f*_*ω*_*ω*〉 for our DNS runs HR1 and HR2.

	*N*	*ν*_*u*_	*ν*_*i*_	*M*	*ξ*	Λ(×*ξ*^2^)	〈*f*_*ω*_*ω*〉	*We*	*L*_*c*_
HR1	2048	10^−35^	2.856	1.0 · 10^−3^	4.42 · 10^−3^	1.0	20	1.7 · 10^−2^	0.75
HR2	4096	10^−35^	2.856	2.5 · 10^−3^	2.21 · 10^−3^	0.8	20	4.3 · 10^−2^	0.38

The forcing wave number is fixed at 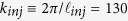, *N*^2^ is the number of collocation points, *ν*_*u*_ is the hyperviscosity, *ν*_*i*_ is the hypoviscosity, *M* is the mobility, parameter, *ξ* sets the scale of the interface width, the surface tension 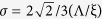, 〈*f*_*ω*_*ω*〉 is the enstrophy-injection rate, which is related to the energy-injection rate [
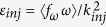
], *L*_*c*_ is the coarsening-arrest length scale [Eq. (3)]. We also conduct a Navier-Stokes run with parameters the same as in run *HR*1 for comparison.
